# Is there a fundamental flaw in Canada’s post-arrival immigrant surveillance system for tuberculosis?

**DOI:** 10.1371/journal.pone.0212706

**Published:** 2019-03-08

**Authors:** Richard Long, Leyla Asadi, Courtney Heffernan, James Barrie, Christopher Winter, Mary Lou Egedahl, Catherine Paulsen, Brenden Kunimoto, Dick Menzies

**Affiliations:** 1 Department of Medicine, Faculty of Medicine and Dentistry, University of Alberta, Edmonton, Alberta, Canada; 2 Department of Radiology and Diagnostic Imaging, Faculty of Medicine and Dentistry, University of Alberta, Edmonton, Alberta, Canada; 3 Montreal Chest Institute, McGill University, Montreal, Quebec, Canada; University of British Columbia, CANADA

## Abstract

**Background:**

New immigrants to Canada with a history of tuberculosis or evidence of old healed tuberculosis on chest radiograph are referred to public health authorities for medical surveillance. This ostensible public health protection measure identifies a subgroup of patients (referrals) who are at very low risk (compared to non-referrals) of transmission.

**Methods:**

To assess whether earlier diagnosis or a different phenotypic expression of disease explains this difference, we systematically reconstructed the immigration and transmission histories from a well-defined cohort of recently-arrived referral and non-referral pulmonary tuberculosis cases in Canada. Incident case chest radiographs in all cases and sequential past radiographs in referrals were re-read by three experts. Change in disease severity from pre-immigration radiograph to incident radiograph was the primary, and transmission of tuberculosis, the secondary, outcome.

**Results:**

There were 174 cohort cases; 61 (35.1%) referrals and 113 (64.9%) non-referrals. Compared to non-referrals, referrals were less likely to be symptomatic (26% vs. 80%), smear-positive (15% vs. 50%), or to have cavitation (0% vs. 35%) or extensive disease (15% vs. 59%) on chest radiograph. After adjustment for referral status, time between films, country-of-birth, age and co-morbidities, referrals were less likely to have substantial changes on chest radiograph; OR 0.058 (95% CI 0.018–0.199). All secondary cases and 82% of tuberculin skin test conversions occurred in contacts of non-referrals.

**Conclusions:**

Phenotypically different disease, and not earlier diagnosis, explains the difference in transmission risk between referrals and non-referrals. Screening, and treating high-risk non-referrals for latent tuberculosis is necessary to eliminate tuberculosis in Canada.

## Introduction

To mitigate the risk of importing tuberculosis (TB) and to protect the public’s health, many countries screen new immigrants for TB. After the Second World War, chest radiography became the standard screening practice [1–4]. However, with increasing calls for TB elimination and more widely available alternate screening methods (i.e. interferon-gamma release assays), this time-honoured radiograph-based screening strategy may at best be insufficient or at worst, creating a false sense of assurance [1, 5–8].

**Fig 1:**
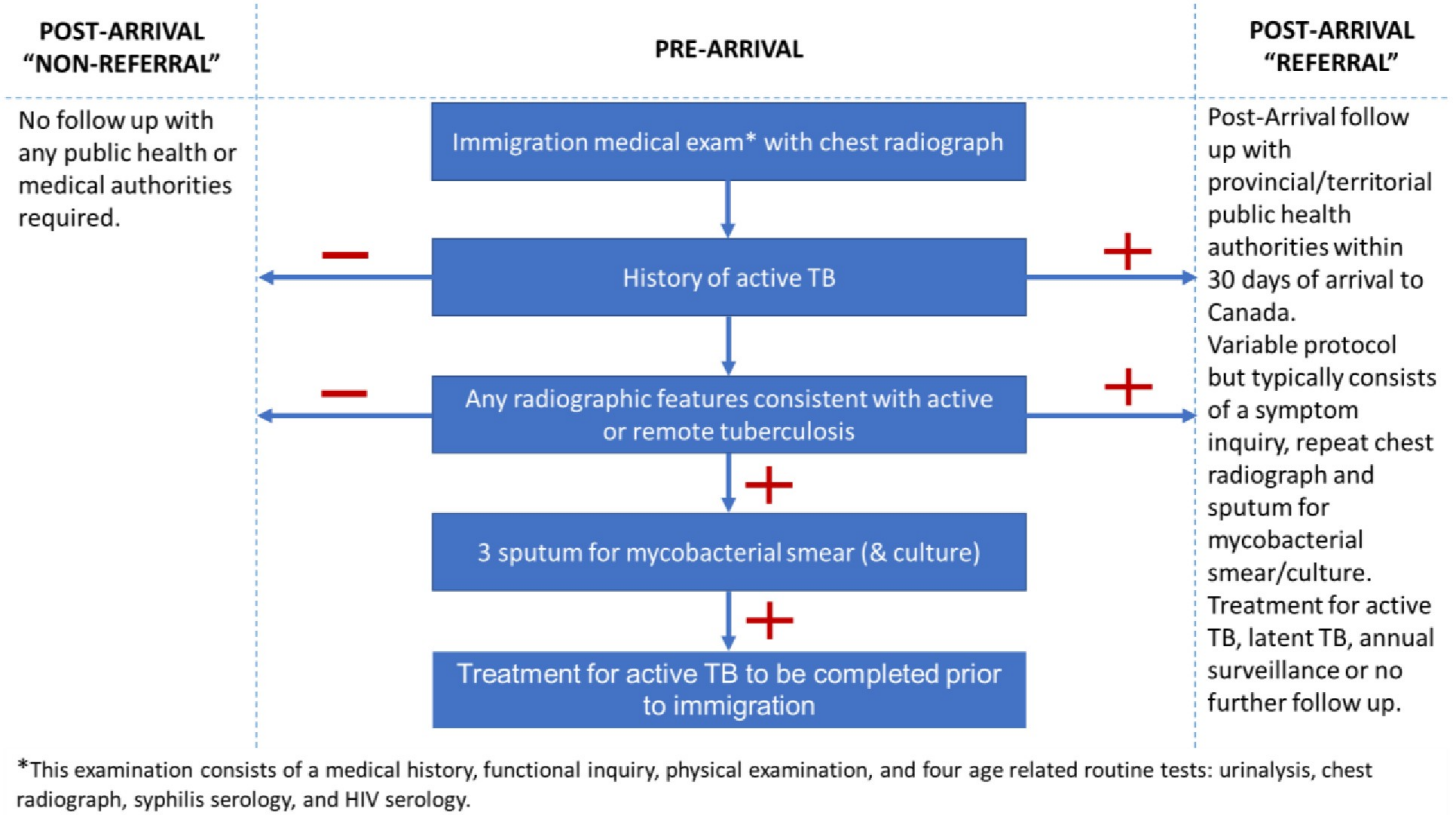
Canada’s pre- and post-arrival immigrant surveillance system for tuberculosis.

To date, Canada’s TB surveillance system has relied on the chest radiograph to i) identify persons with prevalent active TB pre-arrival and ii) identify persons at risk of developing and transmitting TB post-arrival. Applicants with a history of treated TB or evidence of old healed TB on pre-arrival chest radiograph, hereafter “referrals”, undergo follow-up with public health authorities after arrival–similar to Class B referrals in the United States (see Fig 1) [9,10]. Referrals are at higher risk of developing active pulmonary TB [11], but the commonly held belief that they pose a serious public health threat has been challenged [12–14]. New evidence suggests that referrals who develop active TB are far less likely to transmit TB than “non-referrals” [14]. This lower risk of transmission has been assumed to reflect earlier diagnosis of TB among referrals. However, an alternative explanation is that the referral group represents a phenotypically different disease. As early as 1979, Kurt Toman, posited this hypothesis and questioned the merits of routine radiographic screening [15]. Radiographic screening assumes that “TB in adults starts as a rule with a minimal lesion ‘early infiltrate’ that—without treatment—would all develop step by step into advanced, smear-positive tuberculosis. However, studies in populations under surveillance have shown that newly detected, smear-positive TB usually develops fast—ie, without passing through a clinically perceptible initial stage” [15]. That is, the TB infection identified via screening radiography may not develop into active TB disease with the high morbidity or infectiousness most concerning to public health programs. Indeed, the more dangerous, and infectious, smear-positive TB may be more likely to develop in those lacking radiographic evidence of old healed TB.

To explore whether referrals and non-referrals have phenotypically different disease, we examined pre-arrival and incident (at time of TB diagnosis) chest radiographs of referrals and non-referrals who developed active pulmonary TB within two years of arrival to Canada. We hypothesized that chest radiographs among non-referrals would show more rapid disease progression.

## Methods

### Population

This retrospective cohort study was undertaken in Alberta, Canada, where all TB services are provided out of three public health clinics, one clinic in each of the two major cities, Edmonton and Calgary, and one provincial (“virtual”) clinic serving rural Alberta [16]. The provincial clinic is the steward of the TB Registry and receives notifications from Immigration, Refugee, and Citizenship Canada (IRCC) of all immigrants (and some temporary residents) whose admission to the province is conditional on compliance with TB surveillance. These referrals are estimated to be 2–3% of all new immigrants to the province [14, 17].

Over a 160-month period beginning January 1st, 2004, all foreign-born, culture-positive pulmonary TB patients managed out of the Edmonton and Provincial TB Clinics were identified in the TB Registry. These included Canadian citizens, permanent residents, refugees, refugee claimants, and temporary residents. Only patients who were ≥12 years of age on arrival and whose date of diagnosis (start date of treatment) was within 2 years of the date of arrival were eligible for study. The age criterion is based on the age at which a chest radiograph is required at Canada’s immigration medical exam (≥ 11 years), in turn, based on when children begin to develop adult-type (potentially infectious) pulmonary TB [9, 18], plus the period of time that the immigration medical exam (IME) is considered valid (1 year). Members who had undergone an overseas IME were grouped into those who had, and those who had not, been referred. A small subset of the cohort were diagnosed at an in-Canada IME (the processes for which are the same as the overseas IME). These included refugee claimants (asylum seekers) and those seeking an extension to a visitor visa or a change in immigration status. Another small subset had no IME, either overseas or in-Canada.

### Variables of interest

The demographic and clinical characteristics, immigration status and country-of-birth (high vs low incidence) [19], were abstracted from the TB Registry, individual public health, and hospital records. A high incidence country was: i) a country with an average incidence of TB (all forms) in the year of arrival of the case and the two years preceding it, of ≥150 per 100,000 persons (for cases diagnosed in 2017 the average incidence in 2014–2016, the most current incidence data at the time of writing, was used), or ii) any country of sub-Saharan Africa. The number of pre-treatment specimens and the average time to liquid culture positivity of all culture-positive specimens was confirmed in the Provincial Laboratory for Public Health where all mycobacteriology is performed [20]. First-line antituberculosis drug susceptibility testing and DNA fingerprinting was performed on all initial isolates of *Mycobacterium tuberculosis* [21–23]. DNA fingerprinting was performed using either restriction fragment-length polymorphism (RFLP), supplemented in those isolates with five or fewer copies of the insertion sequence *6110* by spoligotyping, or by 24 loci Mycobacterial Interspersed Repetitive Units (MIRU)–Variable Number Tandem Repeats (VNTR) [21–23].

### Primary Outcome: Radiographic progression

For all referrals and non-referrals, posterior-anterior (PA) and lateral (LAT) chest images acquired at the time of diagnosis (the “incident radiographs”) were assembled and re-read independently by three experts (two chest radiologists and a pulmonologist) blinded to the referral status of the patients. For those patients with infiltration localized to, or predominantly in, the upper lung zones, with or without cavitation—a gas filled space within pulmonary consolidation, a mass or a nodule—but with no discernable intrathoracic adenopathy, the radiograph was categorized as “typical” for adult-type pulmonary TB [20]. All other radiographic patterns were categorized as “atypical” for adult-type pulmonary TB. The radiograph was coded as being normal, exhibiting minimal, moderately-advanced, far-advanced, or miliary TB according to criteria established by the US National Tuberculosis and Respiratory Disease Association (see online S1 Appendix) [24]. Inter-reader variability analysis was performed and discrepant readings were resolved by consensus.

All pre-arrival radiographs in referrals were obtained from IRCC; in accordance with Canadian immigration TB screening requirements for the foreign-born, multiple overseas films had been performed in most [9]. The extent of disease on each of these past radiographs was coded as above for the incident case films. For these individuals, sequential overseas, and for referrals with an in-Canada IME only, sequential in-Canada radiographs (still considered “pre-arrival” as they were prior to the incident film), were then reported as being unchanged, subtly changed (a less than minimal change that was considered to be unrelated to projection, technique or composite shadows), minimally changed (an unequivocal increase in extent or density of abnormality that did not result in the “extent of disease” being re-categorized as greater than “minimal”), or substantially changed (an unequivocal increase in extent or density of abnormality that resulted in the “extent of disease” being re-categorized from normal or minimal to moderately-advanced or greater). For non-referrals, whose overseas chest radiographs had been reported as normal by IRCC panel physicians at the time of their IME, we were unable to re-interpret their films. This was because normal films are not shared with the province or territory of destination of the immigrant, nor are they archived by IRCC beyond a period of 2 years. IRCC was, however, able to: i) confirm that their records did indeed show that these non-referral, pre-arrival films had been read as normal, and ii) provide us with the date the film had been performed.

For purposes of reporting on change in the extent of disease on referral and non-referral radiographs, change was dichotomized into “minimal” (unchanged, subtly changed or minimally changed as above) vs. “substantial” (as above). Consensus opinion on change status is reported.

### Secondary outcome: Transmission events

As a secondary analysis, we examined transmission events (defined as tuberculin skin test [TST] conversion in contacts or active TB disease in both contacts and spatiotemporally associated secondary cases) for both groups (see below).

Information on the number, assessment, tuberculin skin test (TST), and disease status of the contacts for each member of the cohort was abstracted from public health records. Complete assessment included a symptom inquiry and TST 8–12 weeks post-final contact with the source case if not already determined to be TST positive, a chest radiograph if determined to be symptomatic or have a positive TST, and sputum for AFB smear and culture if determined to be symptomatic or have an abnormal chest radiograph. TST reactions were defined according to the Canadian TB Standards with a view to prioritizing sensitivity [9]; a new positive TST a reaction of 5 mm or greater; a conversion an increase of at least 6 mm if a previous TST resulted in a reaction of less than 5 mm. If the true positive nature of the TST was in doubt and an interferon gamma release assay (IGRA) was performed, the results of the latter were considered definitive.

The secondary case analysis was performed using molecular and conventional epidemiology, as previously described [14, 20, 25, 26]. For purposes of comparing fingerprints, the comparison dataset began 6 months before the first cohort case diagnosed in 2004 and ended 6 months after the last cohort case diagnosed in 2017. Laboratory cross contamination was systematically excluded in all patients who had a single, smear-negative, culture-positive respiratory specimen [27].

Secondary cases were identified by cross-referencing contact lists of all cohort cases against the Provincial Registry of notified cases in the study years. Secondary cases among contacts were categorized as *Type 1* or *Type 2* based on their conventional and molecular epidemiologic links to cohort cases as follows: *Type 1* are individuals diagnosed with active TB within a transmission window that extends from 6 months before to 24 months after the date of diagnosis of the case to whom they had been identified as a contact, and if they are culture-positive with an isolate of *M*. *tuberculosis* that matched genotypically that of the putative source case. *Type 2* are individuals notified with active TB within the same transmission window who are listed as a contact, but who were culture-negative (mainly children). To account for the possibility that members of the study cohort had incomplete contact lists, secondary cases were searched for among notified cases of TB in the province who were culture-positive, had a genotypically matched isolate of *M*. *tuberculosis*, and were temporally (diagnosed in the same transmission window) and spatially (lived in the same forward-sortation-area—a geographic unit associated with a postal facility from which mail delivery originates—as determined by the first three digits of their postal code) linked to the source case. These were termed *Type 3* secondary cases. Secondary cases who were index cases, i.e. were diagnosed before the date of diagnosis of the cohort case, were only included if they had primary disease.

The transmission window of 30 months was chosen as the risk of disease after infection is highest during this period of time. In addition, it was anticipated that those contacts who were determined to be newly infected but without disease, would be offered treatment of latent TB infection (LTBI) or alternatively, followed over the subsequent 24 months. “Unreported” contacts with LTBI, by virtue of being beyond the reach of preventive measures, were theoretically at greater risk of disease (*Type 3*). In the event that a source case was themselves a secondary case of someone else, transmission events attributed to them were scrutinized for plausibility to ascertain whether their “secondary” cases were not more appropriately attributed to their own source case.

### Statistical analysis

**Fig 2:**
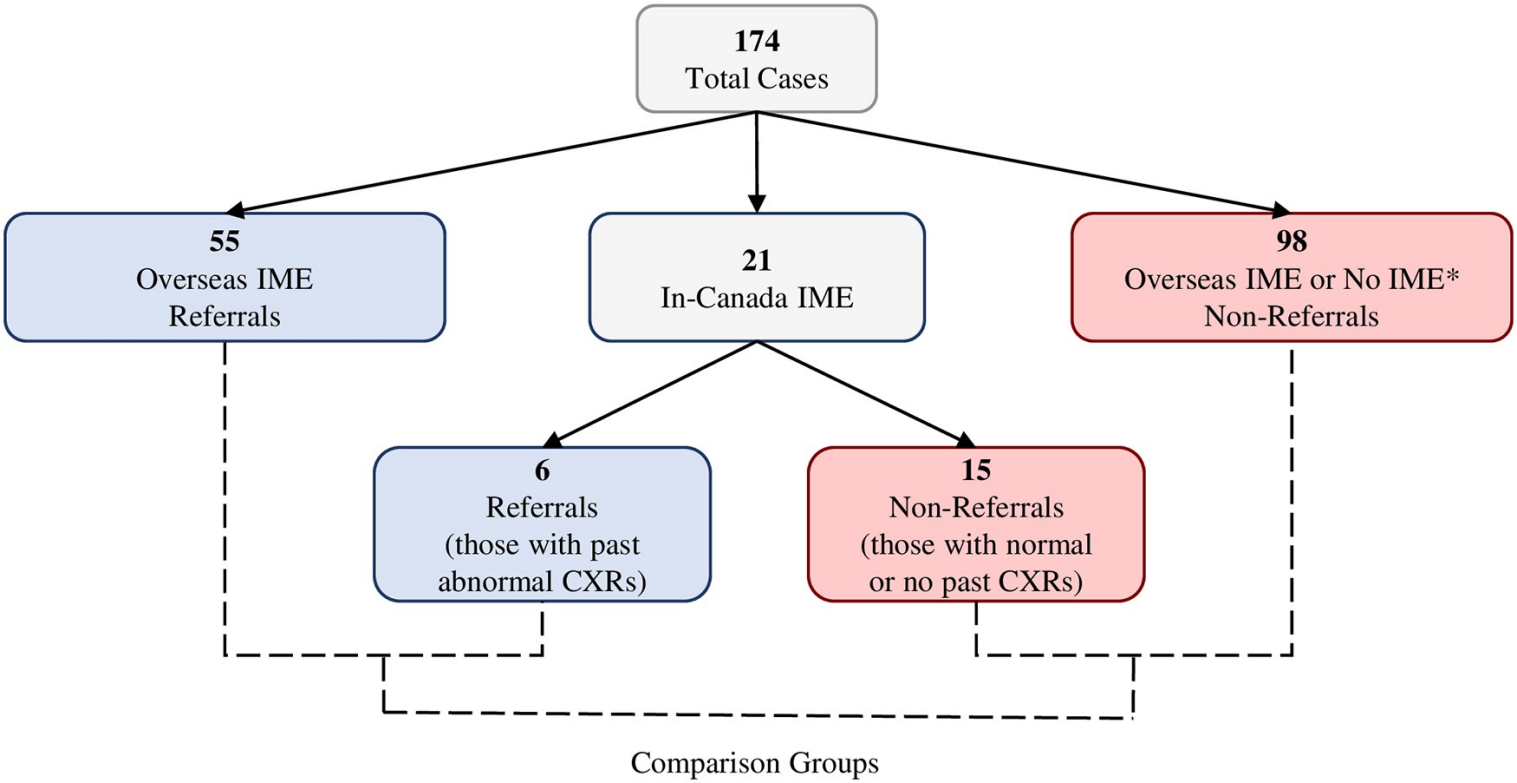
Cohort of recently arrived foreign-born pulmonary tuberculosis cases. Abbreviations: CXR chest x-ray; IME immigration medical examination. * Of the 98 “overseas IME or no IME” non-referrals, 83 had an overseas IME and 15 had neither an overseas nor an in-Canada IME. Of the 83 non-referrals with an overseas IME, 80 had a normal overseas CXR and 3 had an abnormal overseas CXR. † By past CXRs we mean >3 months before their incident case film.

We report generalized kappa statistics to quantify the level of agreement between readers for incident radiographs. The formula proposed by Abraira and Pérez de Vargas was used when weighted categories were necessary [28]. We used multivariate logistic regression to determine the independent association between referral for tuberculosis surveillance (referral status) and degree of chest radiograph progression (minimal vs substantial). We adjusted for variables known to alter the radiographic presentation of pulmonary TB, including, age, HIV status and diabetes [2929–33]. We also adjusted for other potential confounders including “time between films” defined as the number of days between the oldest pre-arrival radiograph and incident radiograph, immigration status (permanent resident or citizen vs refugee), and risk of tuberculosis in country-of-birth (high risk vs lower risk). This main analysis was carried out only on the patients for whom pre-arrival chest radiographs were available (see Fig 2). We also carried out two sensitivity analyses. For the first, we considered referrals who had any instability in their chest radiograph and used the time from their newest pre-arrival radiograph to incident radiograph. Second, we included all referrals and non-referrals, by imputing the average “time between films” of non-referrals without past chest radiographs. We also assumed that these non-referrals would have had “minimal” change between their pre-arrival radiograph and their incident radiograph. Data were analyzed using SAS 9.4. Study approval was obtained from the University of Alberta Health Research Ethics Board (HREB), Panel B, which reviews all non-invasive research projects (PRO-00070701).

## Results

Between January 1, 2004 and April 30, 2017, 174 foreign-born persons ≥ 12 years of age were diagnosed with culture-positive pulmonary TB within 24 months of arrival. Of these, 61 (35.1%) were referrals and 113 (64.9%) were non-referrals. Of the referrals, 6 (9.8%) were diagnosed at an in-Canada IME and had past abnormal in-Canada chest radiographs; of the non-referrals, 15 (13.3%) were diagnosed at an in-Canada IME and had no past chest radiographs (see Fig 2). Most referrals (93.4%) were diagnosed at an initial or one-year follow-up visit; of those that were overseas IME referrals, most (90.6%) were documented to have negative sputum mycobacteriology at their IME. On average, referrals were diagnosed sooner after arrival than non-referrals (mean ± SD 21.6 ± 19.1 vs 47.0 ± 28.5 weeks; median 12.0 vs 41.9 weeks).

Referrals were more likely than non-referrals to be older (mean [±SD] and median age 46.9 ± 18.2; 45 years and 35.3 ± 15.3; 31 years, respectively), to have a past history of TB, and to be asymptomatic, see Table 1. Cough was reported in 12 (19.7%) referrals and 79 (69.9%) non-referrals. Referrals and non-referrals did not differ by sex, co-morbidity status, immigration status, or high vs low incidence country-of-birth. Nobody had dialysis dependent renal failure. The five leading countries of birth of referrals were the Philippines, China, India, Vietnam, and Somalia; of non-referrals the Philippines, India, Ethiopia, Congo, and Somalia.

**Table 1:** Demographic and clinical characteristics of foreign-born pulmonary TB patients by referral status.

Demographic, Clinical and Immigration Characteristics	Total	Referrals n (%)	Non-Referrals n (%)	p-value
**No. Assessed**	174	61	113	
**Age (years)**				
14–64	152	47 (77)	105 (93)	**0·003**
>64	22	14 (23)	8 (7)	
**Sex**				
M	94	35 (57)	59 (52)	0·514
F	80	26 (43)	54 (47)	
**Disease Type**				
New active	150	45 (74)	105 (93)	**0·001**
Relapse/retreatment	24	16 (26)	8 (7)	
**Symptoms**				
Yes	107	16 (26)	91 (81)	**<0·001**
No	67	45 (74)	22 (19)	
**HIV Status**				
Positive	10	2 (3)	8 (7)	0·497
Negative	164	59 (97)	105 (93)	
**Diabetes**				
Yes	19	9 (15)	10 (9)	0·233
No	155	52 (85)	103 (91)	
**Immigration Status**				
Permanent Resident	87	35 (57)	51 (45)	
Temporary resident	71	23 (38)	49 (43)	0·185
Refugee	16	3 (5)	13 (12)	
Country-of-Birth*				
High Incidence	136	53 (87)	96 (85)	1·729
Low Incidence	38	8 (13)	17 (15)	

Abbreviations: M male; F female; HIV human immunodeficiency virus;

* high incidence = i) a country with an average incidence of TB (all forms) in the year of arrival of the case and the two years preceding it, of ≥150 per 100,000 persons (for cases diagnosed in 2017 the average incidence in 2014–2016, the most current incidence data at the time of writing, was used), or ii) any country of sub-Saharan Africa.

Referrals were less likely than non-referrals to be smear-positive and they had longer times to culture-positivity, indicative of lower bacillary loads. There was no difference in drug susceptibility test results, Table 2. Referrals were also less likely to have cavitation or extensive disease (moderately-advanced, far-advanced or miliary) on chest radiograph. Referrals and non-referrals did not differ by incident case chest radiograph category (typical vs. atypical), or laterality (unilateral vs. bilateral). One asymptomatic, smear-negative referral was pregnant and declined an incident case chest radiograph. Six non-referral incident case chest radiographs had been purged and reports alone were available for interpretation. Expert inter-reader agreement was good (see Table 3).

**Table 2:** Mycobacteriologic and incident case chest radiograph (CXR) characteristics in foreign-born, pulmonary tuberculosis patients by referral status.

Characteristic	Total	Referrals n (%)	Non-Referrals n (%)	p-value
**No. Assessed**	174	61	113	
Smear Positive*				
Yes	65	9 (15)	56 (50)	**p <0·001**
No	109	52 (85)	57 (50)	
Time-to-culture-positivity*				
Mean ± SD days		19·8 ± 8·6	13·2 ± 6·9	**p <0·001**
**Drug Resistant**				
Yes	23	8 (13)	15 (13)	0·976
No	151	53 (87)	98 (87)	
**CXR Category**^†^				
Typical	127	43 (72)	84 (74)	
Atypical	34	12 (20)	22 (19)	0·860
Normal	12	5 (8)	7 (6)	
**CXR Laterality**^†^				
Normal	12	5 (8)	7 (6)	
Unilateral	98	38 (63)	60 (53)	0·268
Bilateral	63	17 (28)	46 (41)	
**CXR Cavitation**^†^				
Yes	38	0 (0)	39 (35)	**p <0·001**
No	135	60 (100)	74 (65)	
**CXR Extent of Disease**^†^				
Normal	12	5 (8)	7 (6)	
Minimal	87	46 (77)	40 (35)	
Moderately-advanced	51	8 (13)	44 (39)	**p <0·001**
Far-advanced	19	1 (2)	18 (16)	
Miliary	4	0 (0)	4 (4)	

* Among referrals and non-referrals, respectively, 58 and 80 submitted three, 3 and 13 submitted two, and 0 and 18 submitted one specimen. If multiple pre-treatment specimens were culture-positive, the average time-to-culture positivity was used.

^†^ One referral did not have an incident case chest radiograph. Incident case chest radiographs were performed within 7·8 ± 10·0 (median 2·5) and 5·9 ± 8·5 (median 3·0) days of the date of diagnosis in referrals and non-referrals, respectively (p = 0·20).

**Table 3:** Expert inter-reader variability of incident case diagnostic chest radiograph interpretations.

Patient Group	Expert Reader Interpretation*	Agreement^†^	Kappa Statistic	95% Confidence Interval
**Referrals**	**Category**	Substantial	0.715	[0.543, 0.863]
	**Laterality**	Substantial	0.661	[0.519, 0.826]
	**Cavitation**	n/a^‡^	n/a^‡^	n/a^‡^
	**Extent of Disease**	Substantial	0.751	[0.609, 0.912]
**Non-Referrals**	**Category**	Almost Perfect	0.870	[0.780, 0.945]
	**Laterality**	Substantial	0.775	[0.679, 0.850]
	**Cavitation**	Substantial	0.768	[0.658, 0.843]
	**Extent of Disease**	Substantial	0.727	[0.650, 0.803]
**Both**	**Category**	Almost Perfect	0.814	[0.744, 0.878]
	**Laterality**	Substantial	0.741	[0.666, 0.822]
	**Cavitation**	Substantial	0.767	[0.656, 0.861]
	**Extent of Disease**	Substantial	0.754	[0.694, 0.818]

* See text for definition of chest radiograph category and cavitation; see reference #24 and the S1 Appendix online for definition of extent of disease. Only 10 radiographs (4 in referrals, 6 in non-referrals) required deferring to a process of consensus.

^†^ Kappa statistics were defined as follows: less than 0.00 as poor; 0.00–0.20 as slight; 0.21–0.40 as fair; 0.41–0.60 as moderate; 0.60–0.80 as substantial; 0.81–1.00 as almost perfect.

^‡^ Not available. Because none had cavitation in the referral group, a kappa statistic could not be calculated. Percent agreement was high, with all three readers agreeing that the CXR was non-cavitary in 56 of 60 (93%) referral cases.

**Fig 3:**
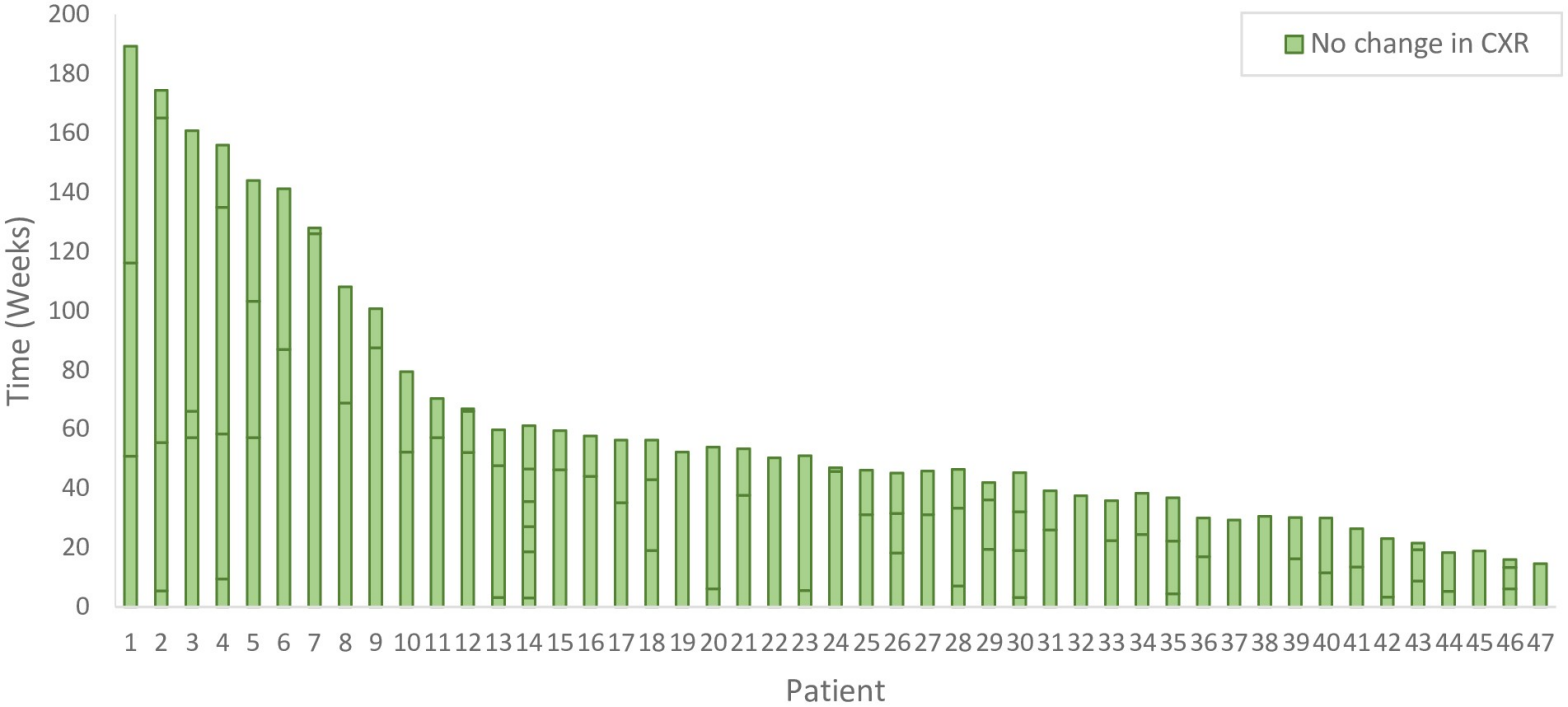
Stable chest radiograph histories in 47 referral cases. Abbreviations: CXR chest x-ray. Each column represents an individual referral; horizontal lines on the columns indicate when CXRs were performed relative to the date of diagnosis. For example, patient #1 had three CXRs at 51, 116 and 189 weeks prior to diagnosis. One referral did not undergo an incident case CXR and is not included.

**Fig 4:**
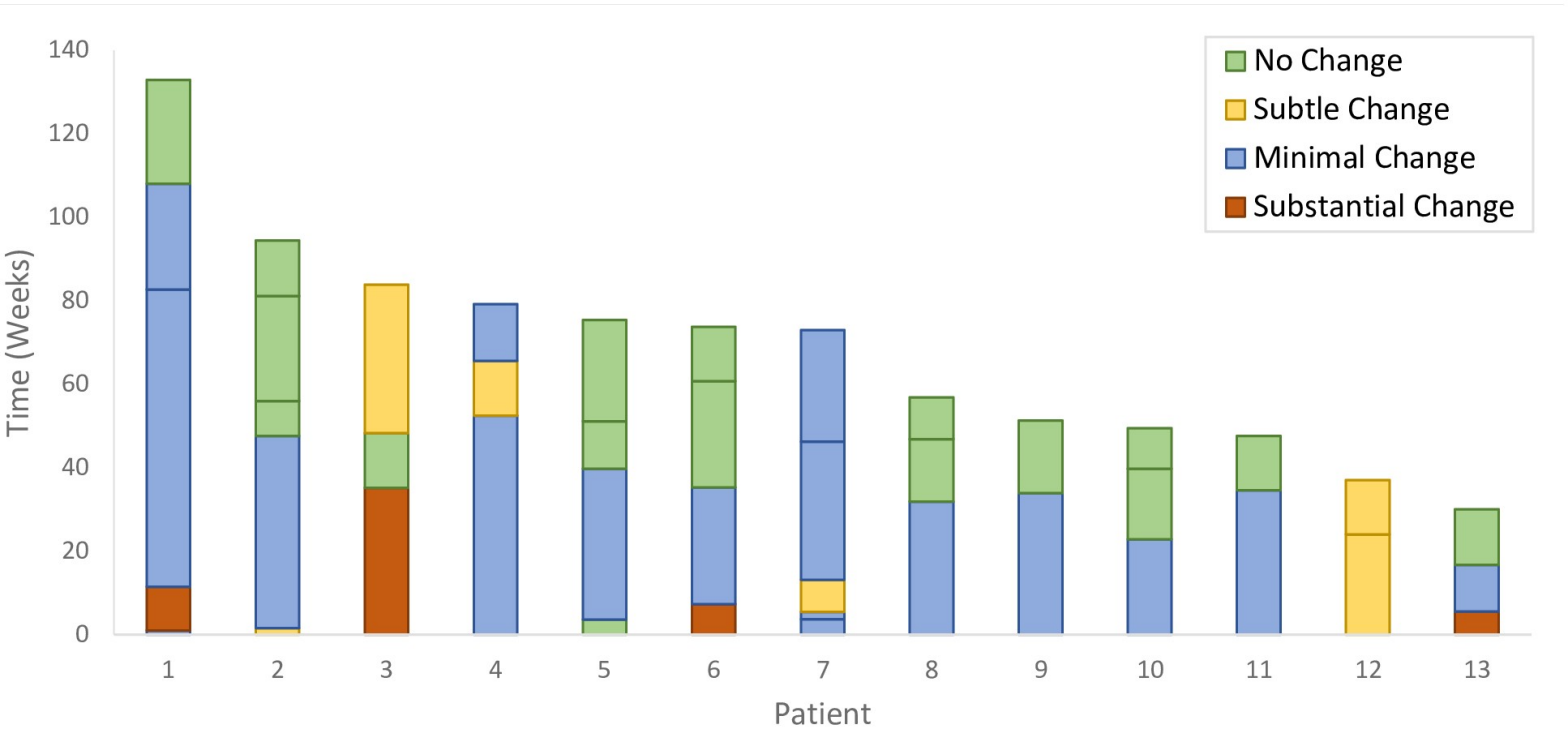
Unstable chest radiograph histories in 13 referral cases. Abbreviations: CXR chest x-ray. Each column represents an individual referral; horizontal lines on the columns indicate when CXRs were performed relative to the date of diagnosis. For example, patient #2 had 5 CXRs at 2, 48, 56, 81 and 94 weeks prior to diagnosis. On the 2^nd^ (at week 81), 3^rd^ (at week 56) and 4^th^ (at week 48) there was no change from the first, but on the next film (at week 2) the independent readers judged there was a minimal change from the 4^th^, and on the next film after that (the incident case film at week 0) there was subtle change from the 5^th^. In these 13 referrals the extent of disease remained unchanged over time in nine (8 remained minimal, 1 remained moderately-advanced). In four, patients #1, 3, 6 and 13, the extent of disease changed from minimal to moderately-advanced.

Among the 60 referrals who had undergone both an incident case as well as one or more past chest radiographs, there was a total of 213 performed radiographs for an average of 3.54 radiographs per referral. In 47 referrals (78.3%), the radiographs were unchanged over an average of 62.2 ± 44.7 weeks (median 47.0 weeks; IQR: 30.6–66.9 weeks), see Fig 3. In 13 referrals (21.7%), the radiograph changed over time. In 9, the final extent of disease remained unchanged; in 4, it progressed from minimal to moderately-advanced, see Fig 4. Among the non-referrals with overseas IMEs, 80 had normal and 3 had abnormal radiographs. In the 80 who had a normal overseas radiograph, the time between the last normal chest radiograph and the incident case film averaged 77.4 ± 35.2 weeks (median 78.1 weeks; IQR: 51.1–100.4 weeks). At diagnosis, the extent of disease was as follows: 5 (6%) normal, 27 (34%) minimal, 31 (39%) moderately-advanced, 13 (16%) far-advanced, 4 (5%) miliary (see example radiographs in S2 Appendix). Thus, 4 (7%) of 60 referrals had substantial changes as compared with 48 (60%) of the 80 non-referrals; adjusted odds ratio 0.058; 95% Confidence Interval (0.018–0.199). This difference persisted in sensitivity analyses (see Table 4).

**Table 4:** The independent association between referral status and degree of chest radiograph (CXR) progression (minimal or substantial).

Referrals vs Non-Referrals with Substantial Radiograph Progression	Unadjusted OR	Adjusted OR
Main analysis (n = 134) (4/54 vs 48/80)*	OR: 0.053 95% CI (0.018–0.162) p<0.0001	OR: 0.058 95% CI (0.018–0.199) p<0.0001
*Sensitivity Analysis 1*: Main analysis using most recent pre-arrival CXR for referrals with unstable CXR (n = 134) (4/54 vs 48/80) ^†^	OR: 0.053 95% CI (0.018–0.162) p<0.0001	OR: 0.047 95% CI (0.013–0.173) p<0.0001
*Sensitivity Analysis 2*: Main analysis, in addition to referrals and non-referrals with in-Canada IMEs (n = 6 and n = 15, respectively), non-referrals with an overseas IME and abnormal CXR (n = 3), and non-referrals with no IME (n = 15) (Total n = 173) (4/60 vs 48/113) ^‡^	0.097, 95% CI 0.033–0.285, p<0.0001	OR: 0.112 95% CI (0.035–0.355) p = 0.0023

* Adjusted for referral status, time from oldest (that is, earliest) pre-arrival CXR to diagnosis CXR “time between films”, immigration status, low or high incidence country-of-birth, age, HIV and diabetes

^†^ Adjustments were as above except that for referrals with any instability in their pre-arrival CXRs, the

“time between films” was time from newest (that is, closest to arrival) pre-arrival CXR to diagnosis CXR

^‡^ Adjustments as per main analysis; for non-referrals without pre-arrival radiograph information (15 with an in-Canada IME; 15 with no IME) or abnormal pre-arrival radiographs (n = 3), we imputed the average “time between films” (from oldest pre-arrival CXR to diagnosis CXR) for non-referrals and also assumed the degree of change was minimal

Two of 174 (1·1%) cohort (both non-referrals) and 53 of 1863 (2·8%) non-cohort case isolates during the study period had not been fingerprinted; most of these unfingerprinted isolates were *M*. *tuberculosis* complex species other than *Mycobacterium tuberculosis*. More non-referrals than referrals had transmission events (40/113 [35%] vs. 12/61 [20%], p<0.04) with the vast majority of transmission events (100% of secondary cases and 81.7% of TST conversions) occurring among contacts of non-referrals (see Table 5 and online S1 Table). Among referral and non-referral converters, only 4 and 16, respectively, had a second TST of ≥ 15mm induration–a result that is known to correlate better with a positive IGRA [34]. There were 16 secondary cases; 13 foreign-born; 3 Canadian-born children of foreign-born parents. All but one was diagnosed within 6 months of the source case.

**Table 5:** Transmission events among contacts of recently arrived, foreign-born, pulmonary TB cases by referral status.

Characteristic	Total	Referrals n (%)	Non-Referrals n (%)
**No. contacts**	2012	352	1660
**No. contacts completely assessed**	1606 (79·8)	299 (84·9)	1307 (78·7)
**No. contacts completely assessed per case**	9·2	4·9	11·6
**No. contacts completely assessed with**:			
Previous Positive TST	91 (5·7)	16 (5·4)	75 (5·7)
New Positive TST	551 (34·3)	118 (39·5)	433 (33·1)
TST Conversion*	82 (5·1)	15 (5·0)	67 (5·1)
Secondary Case	15 (0·9)	0 (0·0)	15 (1·1)
Negative TST	867 (54·0)	150 (50·2)	717 (54·9)

Abbreviation: TST tuberculin skin test

* Of the contacts of referrals that were converters, 4 had a second TST of 15 mm or more; of the contacts of non-referrals that were converters 16 had a second TST of 15 mm or more.

## Discussion

The disease manifested by referrals is relatively benign from an individual and public health perspective [12–14]. Referrals were more likely to be asymptomatic, smear-negative, and to have minimal or no disease on chest radiograph. At the time of diagnosis, the chest radiographic abnormalities in most (78.3%) referrals had been stable for an average of 62.2 weeks and even in referrals whose chest radiographic abnormalities worsened over time (21.7%), the changes were usually subtle or minimal. By contrast, over an average of ~77 weeks, non-referrals progressed from no disease to disease that was often both a threat to themselves and others. Adjusted for relevant demographic and clinical features, non-referrals were much more likely to have substantial changes on their chest radiographs over a similar period. Further, almost all transmission events occurred among contacts of non-referrals.

The observation that immigration referrals are often asymptomatic with minimal and stable radiographic abnormalities was first made by Wang et al in 1991, but its significance was unappreciated at the time [35]. The stability of the chest radiograph together with the absence of symptoms strongly suggests that, despite being diagnosed earlier, the paucibacillary nature of referral’s disease is not a reflection of early diagnosis but rather an accommodation having been reached between the host and pathogen. The immuno-pathologic correlates of this accommodation, once defined, can be expected to impact not only the accuracy estimates for novel TB biomarkers and diagnostics but vaccine development [36, 37]. With respect to non-referrals, while there is abundant literature on the sensitivity and specificity of immunological tests for LTBI (TST and IGRA), the more important measure from an individual perspective and from a public health perspective is the progression rate, the likelihood that a person with a positive test will go on to develop active TB. Currently known risk factors for progression, including the incidence of TB in the country-of-birth, chest radiographic abnormalities (without active TB), moderate or high-risk medical conditions, recent TB contact, immigration status (refugees vs others), and slight but significant variation across immunological tests, are limited in their capacity to predict reactivation [8, 13, 38, 39, 40]. There is an urgent need for a diagnostic test that would predict progression, or alternatively, better identify those at high risk of developing symptomatic and contagious disease.

Strengths of this study include the detailed clinical, radiologic, microbiologic, and transmission information, some of it not readily accessible, such as the referral radiographs, and little of it missing. To our knowledge, this is the first study that compares pre-arrival and post-arrival radiographs and systematically re-interprets pre-arrival films. Shorter time to diagnosis is the main alternate hypothesis for the milder disease in referrals. We therefore not only adjusted for time between radiographs but also undertook sensitivity analyses that considered different definitions of “time between films”. The fact that our finding was unchanged with these analyses lends support to referrals representing a different (less rapidly progressing) phenotype. Weaknesses of our study include its retrospective design and the relatively small sample size; accordingly, it is best understood as a proof-of-concept.

In conclusion, while the value of the overseas IME in detecting prevalent active disease and the need for pre-arrival treatment is undisputed, the value of the post-arrival surveillance system in further protecting the health of the Canadian public is less clear. Referrals, a relatively small group, are at increased risk of disease and ought to continue to be flagged for post-arrival surveillance. However, the disease they manifest constitutes relatively little risk to themselves or others. Non-referrals on the other hand, while a much larger group, appear to include a subgroup of individuals who rapidly progress from LTBI to overt disease that constitutes a risk to both themselves and others. The challenge going forward, is to identify, in a cost-effective manner, this subgroup and to provide them with preventative therapy.

## Supporting information

S1 Appendix Extent of disease. (DOCX)

S2 Appendix Radiographic examples of referral and non-referral patients. (DOCX)

S1 Table Secondary cases among reported and “unreported” contacts of recently arrived foreign-born pulmonary TB cases by referral status. (DOCX)
