# Supplementary material for: Is there a fundamental flaw in Canada’s post-arrival immigrant surveillance system for tuberculosis?
Source: PLoS One. 2019 Mar 8;14(3):e0212706. doi: 10.1371/journal.pone.0212706 (PMC6407769; doi:10.1371/journal.pone.0212706)
Supplement: S1 Appendix — (DOCX) [file pone.0212706.s001.docx]

**S1 Appendix.**

**EXTENT OF DISEASE**

1. **Normal** This classification applies to lesions that cannot be seen on the roentgenogram but are associated with positive cultures for *M. tuberculosis* complex*.*
2. **Minimal** lesions include those that are of slight to moderate density but do not contain demonstrable cavitation. They may involve a small part of one or both lungs, but the total extent, regardless of distribution, should not exceed the volume of lung on one side, which is present above the second chondrosternal junction and the spine of the fourth or the body of the fifth thoracic vertebra. The term minimal is not to be interpreted as minimizing the activity or hazards of the disease in this stage.
3. **Moderately advanced** lesions may be present in one or both lungs, but the total extent should not exceed the following limits: disseminated lesions of slight to moderate density that may extend throughout the total volume of one lung or the equivalent in both lungs; dense and confluent lesions that are limited in extent to one-third the volume of one lung; total diameter of cavitation if present must be less than 4 cm.
4. **Far advanced** is used to describe lesions that are more extensive than moderately advanced.
5. **Miliary** Diffuse micronodular pattern.

REFERENCES (numbers are those from the main manuscript)

1. Falk A, O’Connor JB, Pratt PC, et al. Classification of pulmonary tuberculosis. In: Diagnostic Standards and Classification of Tuberculosis. 12^th^ Edition. New York: *National Tuberculosis and Respiratory Disease Association*; 1969: 68–76.
