# Supplementary material for: Is there a fundamental flaw in Canada’s post-arrival immigrant surveillance system for tuberculosis?
Source: PLoS One. 2019 Mar 8;14(3):e0212706. doi: 10.1371/journal.pone.0212706 (PMC6407769; doi:10.1371/journal.pone.0212706)
Supplement: S2 Appendix — (DOCX) [file pone.0212706.s002.docx]

**S2 Appendix.**

**RADIOGRAPHIC EXAMPLES OF REFERRAL AND NON-REFERRAL PATIENTS**





S1 Fig. Referral. Serial chest radiographs in a 73-year-old male with no past history of pulmonary TB. Radiographs dated March 5, July 12, and October 1, 2012 (incident case film) were performed in-Canada in connection with the extension of a visitor visa. These films were reported as showing stable, typical, unilateral, non-cavitary and minimal disease. This patient was asymptomatic and smear-negative at diagnosis.





S2 Fig. Referral. Serial chest radiographs in an 84-year-old male with a past history of pulmonary TB. Overseas films dated September 11, 2013, June 23, 2014, and May 12, 2015, and the in-Canada incident case film dated June 15, 2016 were reported as showing typical, bilateral, non-cavitary and moderately-advanced disease that was stable over time. This patient was asymptomatic and smear-negative at diagnosis.





S3 Fig. Non-referral. Serial chest radiographs in a 14-year-old female. Overseas films dated February 25, 2009, and December 14, 2010 were reported to be within normal limits. The in-Canada incident case film preformed on March 25, 2012 when the patient was symptomatic and smear-positive was reported as showing typical, bilateral, cavitary and moderately-advanced disease.
