# Supplementary material for: Is there a fundamental flaw in Canada’s post-arrival immigrant surveillance system for tuberculosis?
Source: PLoS One. 2019 Mar 8;14(3):e0212706. doi: 10.1371/journal.pone.0212706 (PMC6407769; doi:10.1371/journal.pone.0212706)
Supplement: S1 Table — (DOCX) [file pone.0212706.s003.docx]

**S1 Table. Secondary cases among reported and “unreported” contacts of recently arrived foreign-born pulmonary TB cases by referral status.**

| **Secondary Case by Type*** | **Total** | **Source Case** | |
| --- | --- | --- | --- |
|  |  | **Referrals**  **n (%)** | **Non-Referrals**  **n (%)** |
| ***Type 1*** | 6 | 0 (0) | 6 (100) |
| ***Type 2*** | 9 | 0 (0) | 9 (100) |
| ***Type 3*** | 1 | 0 (0) | 1 (100) |
| **All Types** | 16 | 0 (0) | 16 (100) |

* *Type 1*: Individuals diagnosed with active TB within a transmission window that extended from 6 months before to 24 months after the date of diagnosis of the putative source case, listed as a contact of the putative source case and culture-positive with an isolate of *M. tuberculosis* that matched genotypically that of the putative source case. *Type 2*: Individuals notified with active TB within the same transmission window, listed as a contact but who were culture-negative (mainly children).
*Type 3*: Individuals who were not reported as a contact but who were culture-positive, had a genotypically matched isolate of *M. tuberculosis*, and were temporally (diagnosed within the same 30 month transmission window) and spatially (lived in the same forward sortation area – a geographical unit associated with postal facility from which mail delivery originates – as determined by the first three digits of their postal code).
Secondary cases diagnosed before the start date of treatment of the putative source case had to have primary disease.
